# Association Between Fruits and Vegetables Consumption and Premature Coronary Artery Disease (PCAD): A Multi‐Center Case Control Study

**DOI:** 10.1002/fsn3.72371

**Published:** 2026-09-29

**Authors:** Noushin Mohammadifard, Sara Aghakhani, Ehsan Zarepur, Alireza Khosravi Farsani, Nahid Azdaki, Nahid Salehi, Masoud Lotfizadeh, Samad Ghaffari, Arsalan Salari, Mostafa Cheraghi, Ahmadreza Assareh, Razieh Hassannejad, Azam Soleimani, Nizal Sarrafzadegan

**Affiliations:** ^1^ Isfahan Cardiovascular Research Center, Cardiovascular Research Institute Isfahan University of Medical Sciences Isfahan Iran; ^2^ Interventional Cardiology Research Center, Cardiovascular Research Institute Isfahan University of Medical Sciences Isfahan Iran; ^3^ Hypertension Research Center, Cardiovascular Research Institute Isfahan University of Medical Sciences Isfahan Iran; ^4^ Iranian Network of Cardiovascular Research (INCVR) Tehran Iran; ^5^ Cardiovascular Diseases Research Center Birjand University of Medical Sciences Birjand Iran; ^6^ Clinical Research Development Unit, Razi Hospital Birjand University of Medical Sciences Birjand Iran; ^7^ Cardiovascular Research Center, Health Institute Kermanshah University of Medical Sciences Kermanshah Iran; ^8^ Department of Community Health Shahrekord University of Medical Sciences Shahrekord Iran; ^9^ Cardiovascular Research Center Tabriz University of Medical Sciences Tabriz Iran; ^10^ Cardiovascular Diseases Research Center, Department of Cardiology, Heshmat Hospital, School of Medicine Guilan University of Medical Sciences Rasht Iran; ^11^ Department of Cardiovascular Research Center, Shahid Rahimi Hospital Lorestan University of Medical Sciences Khorramabad Iran; ^12^ Atherosclerosis Research Center Ahvaz Jundishapur University of Medical Sciences Ahvaz Iran; ^13^ Department of Biostatistics and Epidemiology, School of Health Isfahan University of Medical Sciences Isfahan Iran; ^14^ Cardiac Rehabilitation Research Center, Cardiovascular Research Institute Isfahan University of Medical Sciences Isfahan Iran

**Keywords:** cardiovascular disease, coronary heart disease, diet, fruits, vegetables

## Abstract

Few investigations have examined the association of fruits and vegetables with premature coronary artery disease (PCAD) in the Eastern Mediterranean region. This study aimed to evaluate the association between fruits and vegetables intake and PCAD, as well as its severity. This multi‐centric case–control study was conducted in the framework of the Iran premature coronary artery disease (IPAD) study. Totally, 3249 participants aged < 60 years in men and < 70 years in women, who were referred for angiography to cardiac catheterization and coronary angiography units across 14 cities in Iran representing eight major Iranian ethnicities were examined. Cases were 2068 patients with a stenosis ≥ 75% in at least one vessel or ≥ 50% in the left main cardiac artery. The Control group was 1181 individuals with a normal angiography test. Dietary intake was assessed using the validated semi‐quantitative food frequency questionnaire (FFQ). After full adjustment for confounders, the inverse association with 91%, 96% and 95% PCAD risk reduction was manifest with most frequent fruit consumption (OR: 0.09, 95% CI: 0.06, 0.13), vegetable intake (OR: 0.04, 95% CI: 0.02, 0.06) and total fruits and vegetables consumption (OR: 0.05, 95% CI: 0.03, 0.07), respectively. In both genders, greater intake of fruits, vegetables, and total fruits and vegetables was considerably and negatively associated with the risk of PCAD. Furthermore, the severity of PCAD was inversely correlated with fruits and vegetables intake (*p* < 0.001). We found an inverse relationship between fruits and vegetables consumption with odds of PCAD in Iranian ethnicities. Our findings highlight dietary habits' role in reducing PCAD risk and severity, guiding prevention and clinical care.

## Graphical abstract

A multicenter case–control study of 3249 Iranian adults, including 2068 patients with PCAD and 1181 healthy controls, representing eight Iranian ethnic groups. Dietary intake was assessed using a validated semi‐quantitative food‐frequency questionnaire, and participants were categorized into quartiles according to their consumption of fruits, vegetables, and total fruits and vegetables. Compared with the lowest intake, higher consumption was strongly associated with lower odds of PCAD. The highest intake of total fruits and vegetables was associated with 95% lower odds of PCAD (OR = 0.05, 95% CI: 0.03–0.07), while fruit and vegetable consumption was associated with 91% (OR = 0.09, 95% CI: 0.06–0.13) and 96% (OR = 0.04, 95% CI: 0.02–0.06) lower odds, respectively. Greater intake of fruits, vegetables, and total fruits and vegetables was also inversely associated with PCAD severity (*p* < 0.001). Overall, higher fruit and vegetable consumption was inversely associated with both the odds and severity of PCAD, supporting a fruit‐ and vegetable‐rich dietary pattern as a potentially beneficial component of dietary strategies for PCAD prevention and risk reduction.

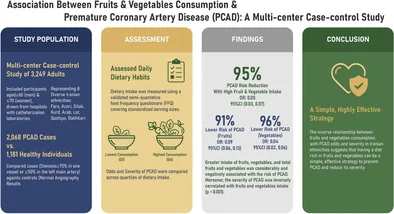

## 1 Introduction

Ischemic heart disease is one of the first causes of mortality and disability‐adjusted life years (DALYs) in adults in Iran and worldwide (Sarrafzadegan and Mohammmadifard 2019; GBD 2019 Diseases and Injuries Collaborators 2020). Although there have been significant advances in the diagnosis and quality of care for patients with cardiovascular disease (CVD), its prevalence among younger age groups is on the rise, which imposes larger costs and burdens on health systems and causes a major public health challenge (Khoja et al. 2021). In recent years, the prevalence of premature coronary artery disease (PCAD) and its mortality rate have increased globally and in Iran (Sarrafzadegan and Mohammmadifard 2019). More than 80% of people with PCAD have at least one modifiable risk factor, including hypertension, diabetes mellitus, obesity, dyslipidemia, psychosocial stress, smoking, unhealthy diet, and physical inactivity (Khoja et al. 2021).

A heart‐healthy diet is a critical component in the prevention and management of CVD. A diet high in saturated and trans fats, cholesterol, and sodium is associated with an increased risk of CVD, while a diet rich in fruits, vegetables, and whole grains is associated with a decreased risk (Miller et al. 2017; Sharifi‐Rad et al. 2020).

Fruits and vegetables are rich in nutrients, which have protective effects on the heart through various mechanisms, including reduction of oxidative stress and inflammation (Bhupathiraju et al. 2013; Lai et al. 2015). Several studies have investigated the association between fruits and vegetables consumption and the risk of CAD and other CVD outcomes. A meta‐analysis of 66 studies found that higher consumption of fruits and vegetables was associated with a reduced risk of coronary heart disease (CHD) (Aune et al. 2017). Another meta‐analysis reported that each additional serving of fruits and vegetables consumed per day was associated with a 4% reduced risk of CHD (Dauchet et al. 2006). Interventional studies have also explored the effects of increasing fruits and vegetables consumption on CVD risk factors (Hartley et al. 2013). Based on American Heart Association (AHA) recommendations, consuming at least 5 servings of fruits and vegetables per day can be useful for prevention of CVD (Krauss et al. 2000). In addition, some studies conducted in Iran have shown that consumption of fruits and vegetables can reduce the incidence and risk of death caused by CVD in adults (Rikhtehgaran et al. 2023). In addition, our previous analyses on the same study evaluated overall dietary patterns in relation to PCAD, in which fruits and vegetables were only part of the healthy pattern (Ghasempour Dabaghi et al. 2024). However, evidence on the association of fruits and vegetables intake and PCAD risk is scarce, especially in the Eastern Mediterranean region (EMR). Hence, we aimed to examine the relationship of fruits and vegetables consumption with occurrence of PCAD and its severity in different ethnicities in Iran.

## 2 Materials and Methods

### 2.1 Study Design and Population

This was a multicenter case–control study on the framework of the IPAD project, which commenced in 2018 (Zarepur et al. 2020). The IPAD study was conducted in diverse ethnicities including Fars, Azari/Turk, Kurd, Arab, Lor, Gilak, Qashqai, and Bakhtiari. The participants' ethnicity was determined based on individuals and their parents' born city. Different cardiac catheterization and coronary angiography units were included across the country. After a comprehensive explanation of the study and obtaining their informed written consent, participants were formally enrolled in the study. This study included adult subjects who were candidates for angiography and aged less than 70 years old for women and less than 60 years old for men and had the same ethnicity as their parents. The criteria for entering the study group were a stenosis in at least one single coronary artery of at least 75% or a stenosis in the left main coronary of at least 50%, while the criteria for entering the control group was a normal coronary artery. Also, CAD severity was defined as the number of stenotic arteries (one, two, or three as well as the left main artery) was intact per previous studies (Özcan et al. 2018). Moreover, patients with a history of confirmed CAD, balloon angioplasty, coronary artery bypass surgery (CABG), or percutaneous coronary intervention, energy intake less than 800 kcal/day or more than 4200 kcal/day, failure to complete any fruits or vegetables questions and more than 20 other food items, or a history of taking herbal supplements in the last 3 months were excluded. Participants with reported total energy intake < 800 or > 4200 kcal/day were excluded, as such values are considered physiologically implausible for adults and likely to reflect substantial misreporting. These cutoffs have been widely used in epidemiological studies employing FFQs to improve data validity.

The sample size in the present analysis was determined by the number of eligible cases and controls enrolled in the IPAD project during the recruitment period (*n* = 3249). A post hoc power calculation indicated that, given this sample size, the study had more than 80% power at a two‐sided *α* of 0.05 to detect odds ratios of approximately 0.60 or lower for the association between the highest versus lowest categories of fruit and vegetable intake and PCAD risk. To ensure the accuracy in diagnosing the disease was the same across centers, all centers adhered to standardized diagnostic criteria for identifying PCAD. Physicians and technicians were trained to ensure consistency in imaging techniques and interpretation, and periodic quality control checks were conducted. Validated imaging equipment and protocols were used across all centers to minimize variability in diagnostic accuracy. Details of the IPAD study were described elsewhere (Zarepur et al. 2020).

This study was approved by the ethics committee of Isfahan University of Medical Sciences (IR.MUI.REC.1396.2.055). A written informed consent was obtained from all patients and the Declaration of Helsinki was considered.

### 2.2 Data Collection

Different cardiac catheterization and coronary angiography units were included across the country, including Askarieh and Shahid Chamran Hospitals in Isfahan city (Fars), Shahid Madani Hospital in Tabriz, Ayatollah Mousavi Hospital in Zanjan and Maragheh city (Azari or Turk), Heshmat Hospital in Rasht (Gilak), Imam Ali Hospital in Kermanshah (Kurd), Shahid Madani Hospital in Khorramabad (Lor), Imam Khomeini Hospital in Ahvaz (Arab), Hajar Hospital in Shahrekord (Bakhtiari), Imam Sajjad Hospital in Yasuj (Qashghaei), Razi Hospital in Birjand (Fars), Afshar Hospital in Yazd (Fars) and Heart Center in Shiraz (Fars). The hospital selection was based on the representativeness and capability of these centers to provide comprehensive care for patients undergoing angiography. Demographic and socioeconomic characteristics including age, sex, education, and marital status; ethnicity; lifestyle including smoking status, physical activity, and dietary intake; and medical history were collected by using a full structured interview by trained health professionals (Rafiemanesh et al. 2020; Mohammadifard et al. 2021).

Body weight was measured using the Seca scale (Seca 750, Seca GmbH & Co, Hamburg, Germany) while participants were barefoot and minimally clothing and height was measured using standard protocols while participants were barefoot and minimally clothing (Khosla and Lowe 1967). Body mass index (BMI) was calculated as weight (kg) divided by the square of height (m^2^). Blood pressure was recorded as the average of two measurements by trained nurses in a sitting position using a mercury sphygmomanometer (Chobanian et al. 2003).

#### 2.2.1 Biochemical Measurements

Fasting blood sample was collected to measure lipid profile and blood glucose levels. Blood specimens underwent centrifugation and were stored at −80°C. Fasting blood sugar (FBS), serum total cholesterol (TC), High‐density lipoprotein cholesterol (HDL‐C), Low‐density lipoprotein cholesterol (LDL‐C), and triglyceride (TG) levels were quantified (Warnick et al. 1982). Dyslipidemia was defined as TC ≥ 200 mg/dL, TG ≥ 150 mg/dL, LDL‐C ≥ 130 mg/dL, and/or HDL‐C < 40 mg/dL for men, and < 50 mg/dL for women, in accordance with the National Cholesterol Education Panel Adult Treatment Panel III (NCEP‐ATP III) guidelines (Lipsy 2003). Diabetes was characterized by FBS levels ≥ 126 mg/dL and/or the use of hypoglycemic medications, as per the American Diabetes Association (ADA) criteria (American Diabetes Association 2018).

#### 2.2.2 Dietary Assessment

Trained interviewers assessed dietary behaviors using a validated semi‐quantitative food frequency questionnaire (FFQ). The validity of FFQ was examined by Pearson correlation coefficients and the De‐attenuated Pearson correlation varied from 0.48 (oils and fats) to 0.65 (sweets) for food groups (all *p* < 0.05). It was 0.49 for fruits and vegetables intake. The reproducibility of FFQ was evaluated by intraclass correlation (ICC) which varied from 0.43 for oils and fats to 0.58 for grains. The ICC was 0.57 for fruits and vegetables intake (re) (Mohammadifard et al. 2021). Each food item was standardized to a common serving size, and respondents were provided with nine response options to indicate frequency of consumption, ranging from rarely to 6 times or more a day. The average daily consumption (g/d) of each food item for each participant was calculated. After that, Nutritionist IV custom software adapted for Iranian food was used to calculate energy and nutrient intake.

### 2.3 Statistical Analysis

Descriptive statistics including mean ± standard deviation (SD) and frequency (percent) were used to express the quantitative and qualitative variables, respectively. We performed one‐way analysis of variance (ANOVA) to compare the mean of quantitative variables among quartiles of fruits and vegetables intake. The normality of data was evaluated by Kolmogorov–Smirnov test and normality plot such as Q‐Q plot. Kruskal‐Wallis test was applied if the normality assumption was not met. Chi‐square test (or Fisher's exact test) was used to compare qualitative variables in different groups. Binary logistic regression analysis was used to assess the relationship between fruits and vegetables consumption and PCAD risk. In addition, the ordinal logistic regression was performed to evaluate the relationship of fruits and vegetables intake and severity of PCAD. The proportional odds assumption was evaluated using the hypothesis test and graphical diagnostics. Although some deviations from the assumption were observed, additional sensitivity analyses including separate binary logistic regression models across severity thresholds as well as a multinomial logistic regression model showed comparable results across alternative modeling strategies. Therefore, the proportional odds model was retained because of its interpretability and parsimonious structure. The lowest quartile was selected as a reference. Odds ratios (ORs) and 95% confidence intervals (CIs) were calculated for each quartile of fruits and vegetables consumption. We used crude and adjusted models to control the effect of potential confounding variables. We used a series of sequential multivariate logistic regression models to examine the robustness of the associations. Model 1 included basic demographic factors (age and sex). Model 2 further adjusted for education and lifestyle variables such as smoking status and physical activity. Model 3 additionally adjusted for clinical and medication history, including hypercholesterolemia, hypertension, diabetes mellitus, aspirin use, family history of CVD, and body mass index (for obesity). Model 4 additionally incorporated dietary intake including energy intake, legumes, fish, grains, non‐hydrogenated vegetable oils (non‐HVOs), red meat, sweets, sugar sweetened beverages (SSBs), dairy, nuts, and seeds. The *p*‐value for trend was calculated by considering fruits and vegetables variables as a continuous variable in all models. Subgroup analyses were performed for different ethnicities to evaluate the potential impact of different ethnicities on the relationship between fruits and vegetables consumption and the risk of PCAD. However, due to the low sample size and sparsity phenomenon, we were unable to apply models and estimate the OR (95% CI) for some ethnicities including Arab, Qashqai, and Bakhtiari. Therefore, we considered merging low‐frequency groups as other. To evaluate the robustness of the primary findings, sensitivity analyses were conducted by excluding participants with major cardiovascular risk factors, including diabetes mellitus, hypertension, and smoking. The logistic regression models were then repeated in the restricted samples, and the resulting estimates were compared with those from the main analysis. The statistical analyses were performed using Statistical Package for Social Sciences (SPSS; version 18.0, SPSS Inc., Chicago, IL, USA) and STATA software, version 14. *p*‐value of less than 0.05 was considered as a significant level.

## 3 Result

In this analysis from IPAD study, 3249 participants were included. Among them, 2068 and 1181 were allocated to the PCAD and control groups, respectively. In our study, 65.8% of the PCAD group and 36.4% of the control group were male. Average age was 54.74 ± 7.1 years for the PCAD group and 51.94 ± 8.3 years for the control group.

In both groups, Individuals with higher intake of total fruits and vegetables were younger (PCAD: *p* < 0.001 and control: *p* = 0.001) and more educated (PCAD: *p* < 0.001 and control: *p* = 0.032). Being male gender was significantly related with higher intake of total fruits and vegetables in PCAD groups (*p* =0.019). Only in PCAD group, the higher consumption of fruits and vegetables was significantly associated with greater BMI (*p* = 0.036). In addition, in control group, the higher consumption of fruits and vegetables was significantly associated with being more physically active (*p* = 0.001) (Table 1).

**TABLE 1:** Basic characteristics of subjects with and without premature coronary artery disease based on quartiles of total fruits and vegetables consumption.

Characteristic	PCAD					Control				
	Q1 < 343.05	343.05 ≤ Q2 < 474.07	474.07 ≤ Q3 < 613.42	Q4 ≥ 613.42	*p*	Q1 < 343.05	343.05 ≤ Q2 < 474.07	474.07 ≤ Q3 < 613.42	Q4 ≥ 613.42	*p*
	*N* = 744	*N* = 642	*N* = 369	*N* = 313		*N* = 68	*N* = 170	*N* = 444	*N* = 499	
Median	279.52	402.89	526.93	781.94		292.70	426.62	545.32	733.02	
Age (years)	55.8 ± 7.1	54.6 ± 7.1	54.0 ± 7.1	53.1 ± 7.2	< 0.001^a^	53.9 ± 6.8	52.2 ± 9.8	52.7 ± 8.0	50.8 ± 8.2	0.001^b^
Sex (male) *n* (%)	457 (61.4)	436 (67.9)	255 (69.1)	212 (67.7)	0.019^c^	19 (27.9)	61 (35.9)	162 (36.5)	188 (37.7)	0.480^c^
Ethnicity (%)					< 0.001^c^					< 0.001^c^
Fars	289 (38.8)	363 (56.5)	232 (62.9)	185 (59.1)		29 (42.6)	81 (47.6)	272 (61.3)	287 (57.5)	
Azari	81 (10.9)	76 (11.8)	38 (10.3)	27 (8.6)		4 (5.9)	13 (7.6)	36 (8.1)	31 (6.2)	
Gilak	126 (16.9)	34 (5.3)	9 (2.4)	5 (1.9)		11 (16.2)	21 (12.4)	24 (5.4)	10 (2)	
Kurd	94 (12.6)	59 (9.2)	23 (6.2)	18 (5.8)		12 (17.6)	31 (18.2)	66 (14.9)	67 (13.4)	
Arab	19 (2.6)	15 (2.3)	15 (4.1)	17 (5.4)		0 (0)	3 (1.8)	7 (1.6)	21 (4.2)	
Lor	72 (9.7)	34 (5.3)	18 (4.9)	22 (7)		1 (1.5)	2 (1.2)	10 (2.3)	13 (2.6)	
Qaghqaei	26 (3.5)	25 (3.9)	11 (3)	11 (3.5)		5 (7.4)	8 (4.7)	11 (2.5)	32 (6.4)	
Bakhtiari	37 (5)	36 (5.6)	23 (6.2)	28 (8.9)		6 (8.8)	11 (6.5)	18 (4.1)	38 (7.6)	
Smoking, *n* (%)	325 (43.8)	283 (44.1)	148 (40.1)	145 (46.5)	0.400^c^	13 (19.1)	40 (23.5)	105 (23.6)	116 (23.2)	0.874^c^
Education (years), *n* (%)					< 0.001^c^					0.032^c^
< 8	554 (74.5)	413 (64.3)	241 (65.5)	198 (63.3)		59 (86.8)	131 (77.1)	323 (72.9)	353 (70.7)	
9–12	123 (16.5)	138 (21.5)	85 (23.1)	71 (22.7)		9 (13.2)	30 (17.6)	83 (18.7)	94 (18.8)	
> 12	67 (9)	91 (14.2)	42 (11.4)	44 (14.1)		0 (0)	9 (5.3)	37 (8.4)	52 (10.4)	
Aspirin use, *n* (%)	140 (18.9)	107 (16.8)	56 (15.6)	64 (20.6)	0.275^c^	30 (44.1)	81 (48.2)	208 (47.1)	239 (49)	0.862^c^
Hypertension, *n* (%)	330 (44.8)	304 (47.9)	159 (44)	126 (41.6)	0.298^c^	29 (43.3)	67 (39.9)	188 (42.8)	192 (39.2)	0.682^c^
Diabetes mellitus, *n* (%)	282 (42.3)	212 (38.8)	107 (33.2)	94 (35.3)	0.032^c^	16 (26.2)	48 (31.6)	94 (23.6)	109 (25.2)	0.286^c^
Hypercholesterolemia, *n* (%)	269 (38.5)	234 (38.7)	123 (35.5)	108 (38)	0.779^c^	28 (42.4)	65 (40.4)	163 (37.7)	166 (35.1)	0.492^c^
Total physical activity METS (hour/week)	31.8 ± 63.0	32.0 ± 49.9	28.8 ± 48.3	38.7 ± 66.6	0.320^b^	33.8 ± 74.6	26.7 ± 53.1	31.4 ± 75.2	41.1 ± 82.6	0.001^b^
BMI (kg/m^2^)	27.6 ± 4.5	28.0 ± 4.5	28.3 ± 4.5	28.4 ± 4.7	0.036^b^	28.3 ± 6.4	28.6 ± 5.1	28.7 ± 4.9	28.8 ± 5.2	0.861^a^

Abbreviations: BMI, body max index; PCAD, premature coronary artery disease.

^a^ Obtained by ANOVA.

^b^ Obtained by Kruskal–Wallis test.

^c^ Obtained by chi‐squared test.

Higher consumption of total fruits and vegetables was significantly associated with higher intake of energy and nutrients including carbohydrate, protein, total fat, saturated fatty acids (SFA), monounsaturated fatty acids (MUFA), polyunsaturated fatty acids (PUFA), and fiber in both groups (*p* < 0.001). Moreover, subjects in both groups with higher intake of total fruits and vegetables had more consumption of legumes, fish, refined grains, non‐HVO, red meat, sweets, low‐fat dairy, nuts, and seeds (All *p* < 0.01). While higher consumption of total fruits and vegetables was only with whole grains in the PCAD group (*p* = 0.001) and with high‐fat dairy and SSBs intake in the control group (*p* < 0.001 and *p* = 0.044, respectively). HVOs intake had not significantly relationship with total fruits and vegetables consumption in both groups (Table 2).

**TABLE 2:** Dietary intake in subjects with and without premature coronary artery disease based on quartiles of total fruits and vegetables consumption.

Nutrients and food groups	PCAD					Control				
	Q1 < 343.05	343.05 ≤ Q2 < 474.07	474.07 ≤ Q3 < 613.42	Q4 ≥ 613.42	*p* ^a^	Q1 < 343.05	343.05 ≤ Q2 < 474.07	474.07 ≤ Q3 < 613.42	Q4 ≥ 613.42	*p* ^a^
	Mean ± SD	Mean ± SD	Mean ± SD	Mean ± SD		Mean ± SD	Mean ± SD	Mean ± SD	Mean ± SD	
Energy (kcal/day)	1828.2 ± 734.0	2005.5 ± 763.2	2152.5 ± 767.7	2597.3 ± 920.3	< 0.001	1405.6 ± 584.5	1692.8 ± 611.0	2012.2 ± 662.4	2326.9 ± 868.2	< 0.001
Carbohydrate (g/day)	240.9 ± 107.4	264.7 ± 120.8	272.4 ± 112.2	326.6 ± 119.9	< 0.001	172.1 ± 87.6	205.9 ± 89.2	249.2 ± 103.0	283.2 ± 119.5	< 0.001
Protein (g/day)	71.1 ± 30.8	78.7 ± 30.9	86.5 ± 31.2	106.5 ± 41.6	< 0.001	55.3 ± 24.6	67.6 ± 26.1	81.0 ± 29.2	97.6 ± 43.4	< 0.001
Total fat (g/day)	73.9 ± 37.9	79.4 ± 34.3	89.2 ± 36.1	109.0 ± 55.0	< 0.001	62.4 ± 30.9	75.3 ± 31.0	86.6 ± 30.7	100.2 ± 40.4	< 0.001
SFA (g/day)	29.8 ± 17.7	31.4 ± 16.2	32.8 ± 15.3	42.0 ± 25.9	< 0.001	20.8 ± 13.9	23.6 ± 10.3	28.9 ± 11.4	36.2 ± 17.7	< 0.001
MUFA (g/day)	23.1 ± 13.2	24.3 ± 11.9	26.4 ± 12.1	32.1 ± 17.6	< 0.001	19.4 ± 11.6	22.7 ± 10.0	25.7 ± 10.4	29.4 ± 12.7	< 0.001
PUFA (g/day)	17.6 ± 10.3	19.8 ± 9.5	24.4 ± 11.7	28.0 ± 14.3	< 0.001	18.0 ± 8.7	23.3 ± 11.2	25.3 ± 10.6	28.7 ± 13.3	< 0.001
Fiber (g/day)	17.1 ± 7.4	20.7 ± 8.4	22.1 ± 8.7	29.7 ± 10.8	< 0.001	13.6 ± 5.8	16.7 ± 6.5	20.5 ± 7.8	26.2 ± 10.4	< 0.001
Food groups (g/day)										
Fruits and vegetables	260.1 ± 69.1	403.5 ± 37.1	531.6 ± 38.8	825.9 ± 230.0	< 0.001	275.0 ± 58.3	422.2 ± 36.8	544.0 ± 40.6	812.2 ± 222.3	< 0.001
Fruits	102.9 ± 54.8	184.8 ± 45.5	209.1 ± 50.8	347.9 ± 138.9	< 0.001	78.3 ± 56.5	174.3 ± 59.9	257.4 ± 58.7	343.2 ± 108.7	< 0.001
Vegetables	159.8 ± 48.8	219.1 ± 45.4	292.6 ± 53.7	505.1 ± 173.4	< 0.001	209.0 ± 62.7	250.3 ± 53.1	287.2 ± 55.3	469.0 ± 186.8	< 0.001
Legumes	30.8 ± 34.4	41.6 ± 33.5	60.6 ± 34.5	92.8 ± 78.5	< 0.001	48.5 ± 37.6	58.3 ± 40.0	66.6 ± 37.1	93.3 ± 83.8	< 0.001
Fish	6.7 ± 9.9	10.8 ± 12.7	17.5 ± 22.5	26.7 ± 39.4	< 0.001	3.6 ± 8.4	12.1 ± 16.1	16.9 ± 20.7	23.8 ± 32.8	< 0.001
Whole grains	110.5 ± 123.6	102.5 ± 125.7	122.3 ± 133.3	130.5 ± 138.2	0.001	113.5 ± 107.4	121.8 ± 129.4	142.8 ± 158.4	124.5 ± 141.1	0.364
Refine grains	228.0 ± 157.9	239.0 ± 163.5	239.5 ± 162.0	278.2 ± 188.5	0.001	171.4 ± 121.0	204.2 ± 141.8	231.2 ± 153.4	245.0 ± 174.0	< 0.001
Non‐HVO	14.5 ± 14.5	15.3 ± 12.2	20.7 ± 15.0	24.5 ± 21.1	< 0.001	15.8 ± 14.1	21.9 ± 16.0	22.2 ± 15.0	24.2 ± 17.2	< 0.001
HVO	6.4 ± 13.2	5.7 ± 10.4	4.5 ± 9.3	5.1 ± 13.1	0.463	3.5 ± 5.2	2.3 ± 4.3	3.1 ± 5.5	2.6 ± 4.9	0.156
Red meat	32.6 ± 47.0	36.5 ± 38.7	37.3 ± 36.0	47.5 ± 61.1	< 0.001	17.5 ± 26.8	20.6 ± 25.6	29.1 ± 28.4	37.6 ± 43.2	< 0.001
Sweets	17.7 ± 25.5	21.9 ± 33.8	19.2 ± 27.8	22.8 ± 29.5	0.002	4.8 ± 7.5	9.8 ± 13.9	16.2 ± 26.3	19.4 ± 29.4	< 0.001
SSBs	64.0 ± 119.8	62.9 ± 118.2	56.9 ± 110.6	49.3 ± 75.9	0.921	25.6 ± 41.5	36.4 ± 113.1	42.2 ± 113.0	41.4 ± 80.7	0.044
Low fat dairy	201.4 ± 143.5	230.4 ± 164.7	318.2 ± 192.5	380.4 ± 257.0	< 0.001	185.3 ± 148.6	211.7 ± 188.8	229.4 ± 181.5	300.0 ± 263.9	< 0.001
High fat dairy	82.0 ± 105.6	87.1 ± 110.0	88.5 ± 117.7	98.4 ± 132.2	0.195	56.7 ± 116.6	48.1 ± 72.0	64.1 ± 91.9	82.6 ± 110.2	< 0.001
Nuts and seeds	9.5 ± 12.4	12.7 ± 14.3	21.4 ± 25.4	21.6 ± 20.4	< 0.001	20.0 ± 21.6	26.1 ± 27.2	26.5 ± 26.8	30.5 ± 32.1	0.018

Abbreviations: HVO, hydrogenated vegetables oil; MUFA, monounsaturated fatty acid; PCAD, premature coronary artery disease; PUFA, polyunsaturated fatty acid; SD, standard deviation; SFA, saturated fatty acids; SSB, sugar sweetened beverage.

^a^ Obtained by Kruskal–Wallis test.

The multivariate‐adjusted ORs and 95% CI for PCAD risk within quartiles of fruits, vegetables, and total fruits and vegetables were shown in Tables 3, 4, 5, respectively. In the crude model for total fruits, a significant risk reduction for PCAD was observed in the third and fourth quartiles compared to the first one (OR: 0.25, 95% CI: 0.19, 0.31 and OR: 0.12, 95% CI: 0.09, 0.15, respectively; *p* for trend < 0.001) in the total population. After adjustment for potential confounders in different models, the association was strengthened in the 3rd and 4th quartiles compared to the first quartile (OR: 0.23, 95% CI: 0.17, 0.32 and OR: 0.09, 95% CI: 0.06, 0.13, respectively; *p* for trend < 0.001). Moreover, compared to the first quartile, those in the 3rd and 4th quartiles had a decreased risk of PCAD in diverse ethnicities including Azari, Fars, Gilak, Kurd, and others in crude and adjusted models (Table 3).

**TABLE 3:** Odds ratio (95% confidence interval) of premature coronary artery disease risk in different ethnicities according to quartile of fruits consumption.

Ethnicity/Model	OR (95% CI)				*p* for trend
	Q1 < 146.01	146.01 ≤ Q2 < 210.97	210.97 ≤ Q3 < 278.41	Q4 ≥ 278.41	
*Total*					
Median	87.43	176.79	240.53	338.22	
Crude	1	1.04 (0.80, 1.34)	0.25 (0.19, 0.31)	0.12 (0.09, 0.15)	< 0.001
Model 1	1	0.94 (0.71, 1.23)	0.24 (0.18, 0.30)	0.11 (0.08, 0.14)	< 0.001
Model 2	1	0.92 (0.70, 1.21)	0.24 (0.18, 0.30)	0.11 (0.08, 0.14)	< 0.001
Model 3	1	0.89 (0.65, 1.21)	0.24 (0.18, 0.31)	0.10 (0.08, 0.14)	< 0.001
Model 4	1	0.83 (0.59, 1.17)	0.23 (0.17, 0.32)	0.09 (0.06, 0.13)	< 0.001
*Azari*					
Median	88.27	179.73	241.55	306.87	
Crude	1	1.00 (0.36, 2.73)	0.14 (0.06, 0.33)	0.08 (0.03, 0.21)	< 0.001
Model 1	1	1.25 (0.43, 3.56)	0.15 (0.06, 0.37)	0.08 (0.02, 0.21)	< 0.001
Model 2	1	1.29 (0.44, 3.71)	0.16 (0.06, 0.38)	0.08 (0.03, 0.22)	< 0.001
Model 3	1	1.54 (0.40, 5.91)	0.12 (0.04, 0.38)	0.08 (0.02, 0.31)	< 0.001
Model 4	1	1.40 (0.18, 10.74)	0.06 (0.01, 0.50)	0.02 (0.01, 0.29)	< 0.001
*Fars*					
Median	115.98	176.91	240.56	336.02	
Crude	1	1.43 (0.97, 2.09)	0.31 (0.22, 0.43)	0.16 (0.11, 0.22)	< 0.001
Model 1	1	1.22 (0.80, 1.83)	0.29 (0.20, 0.42)	0.15 (0.10, 0.21)	< 0.001
Model 2	1	1.21 (0.80, 1.83)	0.30 (0.20, 0.42)	0.15 (0.10, 0.21)	< 0.001
Model 3	1	1.23 (0.75, 2.01)	0.31 (0.20, 0.47)	0.14 (0.09, 0.21)	< 0.001
Model 4	1	1.18 (0.69, 2.04)	0.30 (0.18, 0.48)	0.14 (0.08, 0.23)	< 0.001
*Gilak*					
Median	55.27	171.90	219.95	299.05	
Crude	1	0.27 (0.12, 0.57)	0.08 (0.03, 0.20)	0.08 (0.02, 0.36)	< 0.001
Model 1	1	0.26 (0.11, 0.57)	0.11 (0.04, 0.26)	0.10 (0.02, 0.44)	< 0.001
Model 2	1	0.28 (0.12, 0.63)	0.11 (0.04, 0.29)	0.12 (0.02, 0.56)	< 0.001
Model 3	1	0.23 (0.09, 0.59)	0.14 (0.05, 0.38)	0.09 (0.02, 0.47)	< 0.001
Model 4	1	0.73 (0.17, 3.10)	0.04 (0.01, 0.25)	0.08 (0.01, 0.18)	< 0.001
*Kurd*					
Median	88.33	178.64	241.48	400.89	
Crude	1	0.98 (0.51, 1.88)	0.26 (0.14, 0.48)	0.04 (0.02, 0.09)	< 0.001
Model 1	1	0.96 (0.47, 1.95)	0.22 (0.11, 0.43)	0.04 (0.01, 0.09)	< 0.001
Model 2	1	0.94 (0.46, 1.93)	0.21 (0.10, 0.41)	0.04 (0.01, 0.08)	< 0.001
Model 3	1	1.37 (0.60, 3.15)	0.21 (0.10, 0.45)	0.04 (0.02, 0.11)	< 0.001
Model 4	1	1.30 (0.39, 4.33)	0.14 (0.04, 0.47)	0.02 (0.01, 0.07)	< 0.001
*Others*					
Median	86.3	176.94	241.32	361.90	
Crude	1	0.95 (0.52, 1.76)	0.27 (0.15, 0.47)	0.11 (0.06, 0.18)	< 0.001
Model 1	1	0.90 (0.47, 1.72)	0.29 (0.16, 0.54)	0.11 (0.06, 0.19)	< 0.001
Model 2	1	0.88 (0.45, 1.69)	0.30 (0.16, 0.56)	0.10 (0.06, 0.18)	< 0.001
Model 3	1	0.56 (0.26, 1.20)	0.27 (0.13, 0.58)	0.07 (0.03, 0.14)	< 0.001
Model 4	1	0.51 (0.21, 1.21)	0.35 (0.15, 0.83)	0.06 (0.02, 0.16)	< 0.001

*Note:* Model 1: Age and sex. Model 2: Model 1 + education, smoking (never/ex‐smoker/current smoker), physical activity (METS/min per week). Model 3: Model 2 + hypercholesterolemia (yes/no), hypertension (yes/no), diabetes mellitus (yes/no), aspirin use (yes/no), family history of cardiovascular disease (yes/no) and body mass index (kg/m^2^). Model 4: Model 3 + intake of energy (kcal/day), legumes, fish, grains, non‐hydrogenated vegetable oils, red meat, sweets, sugar sweetened beverages, dairy, nuts and seeds.

In total population, compared to lowest quartile, any higher intake of vegetables had an inverse relationship with lower PCAD occurrence in the crude model (OR: 0.07, 95% CI: 0.04, 0.10, OR: 0.04, 95% CI: 0.02, 0.06 and OR: 0.03, 95% CI: 0.01, 0.04 across quartiles 2, 3 and 4 compared to the first one, respectively; *p* for trend < 0001). Similar results were observed after adjustment for confounders in different models (OR: 0.08, 95% CI: 0.05, 0.13, OR: 0.06, 95% CI: 0.03, 0.09 and OR: 0.04, 95% CI: 0.02, 0.06 based on quartiles 2, 3 and 4 against the first quartile, respectively; *p* for trend < 0.001). Similarly, in different ethnicities, those with any increase in vegetable intake had a reduction in PCAD risk in crude and all adjusted models (Table 4).

**TABLE 4:** Odds ratio (95% confidence interval) of premature coronary artery disease risk in different ethnicities according to quartile of vegetables consumption.

Ethnicity/Model	OR (95% CI)				*p* for trend
	Q1 < 192.06	192.06 ≤ Q2 < 254.35	254.35 ≤ Q3 < 345.24	Q4 ≥ 345.24	
*Total*					
Median	157.01	220.71	285.81	453.40	
Crude	1	0.07 (0.04, 0.10)	0.04 (0.02, 0.06)	0.03 (0.01, 0.04)	< 0.001
Model 1	1	0.07 (0.04, 0.10)	0.04 (0.02, 0.06)	0.03 (0.02, 0.05)	< 0.001
Model 2	1	0.07 (0.04, 0.10)	0.04 (0.02, 0.06)	0.03 (0.02, 0.04)	< 0.001
Model 3	1	0.06 (0.04, 0.09)	0.04 (0.03, 0.06)	0.03 (0.02, 0.05)	< 0.001
Model 4	1	0.08 (0.05, 0.13)	0.06 (0.03, 0.09)	0.04 (0.02, 0.06)	< 0.001
*Azari*					
Median	155.36	216.56	285.73	419.97	
Crude	1	0.10 (0.02, 0.41)	0.04 (0.01, 0.17)	0.02 (0.01, 0.10)	< 0.001
Model 1	1	0.08 (0.01, 0.37)	0.04 (0.01, 0.17)	0.03 (0.01, 0.12)	< 0.001
Model 2	1	0.08 (0.01, 0.37)	0.03 (0.01, 0.16)	0.03 (0.01, 0.12)	< 0.001
Model 3	1	0.08 (0.01, 0.65)	0.03 (0.01, 0.23)	0.02 (0.01, 0.19)	< 0.001
Model 4	1	0.16 (0.01, 1.98)	0.05 (0.01, 0.63)	0.04 (0.01, 0.59)	< 0.001
*Fars*					
Median	161.19	220.71	283.71	462.88	
Crude	1	0.06 (0.03, 0.11)	0.04 (0.02, 0.06)	0.03 (0.01, 0.04)	< 0.001
Model 1	1	0.06 (0.03, 0.11)	0.04 (0.02, 0.06)	0.03 (0.01, 0.05)	< 0.001
Model 2	1	0.06 (0.03, 0.11)	0.04 (0.02, 0.06)	0.03 (0.01, 0.05)	< 0.001
Model 3	1	0.05 (0.03, 0.11)	0.04 (0.02, 0.08)	0.03 (0.01, 0.06)	< 0.001
Model 4	1	0.07 (0.04, 0.15)	0.06 (0.03, 0.12)	0.04 (0.02, 0.08)	< 0.001
*Gilak*					
Median	151.60	219.69	277.03	436.27	
Crude	1	0.09 (0.28, 0.26)	0.05 (0.01, 0.15)	0.02 (0.01, 0.08)	< 0.001
Model 1	1	0.10 (0.03, 0.30)	0.06 (0.01, 0.20)	0.03 (0.01, 0.10)	< 0.001
Model 2	1	0.09 (0.02, 0.29)	0.07 (0.01, 0.23)	0.02 (0.01, 0.08)	< 0.001
Model 3	1	0.05 (0.01, 0.26)	0.03 (0.01, 0.17)	0.01 (0.01, 0.08)	< 0.001
Model 4	1	0.04 (0.01, 0.31)	0.02 (0.01, 0.20)	0.01 (0.001, 0.08)	< 0.001
*Kurd*					
Median	159.64	222.90	286.04	424.69	
Crude	1	0.05 (0.02, 0.13)	0.03 (0.01, 0.08)	0.23 (0.01, 0.06)	< 0.001
Model 1	1	0.05 (0.02, 0.14)	0.03 (0.01, 0.08)	0.03 (0.01, 0.08)	< 0.001
Model 2	1	0.05 (0.01, 0.13)	0.03 (0.01, 0.08)	0.03 (0.01, 0.07)	< 0.001
Model 3	1	0.05 (0.02, 0.15)	0.03 (0.01, 0.09)	0.04 (0.01, 0.11)	< 0.001
Model 4	1	0.08 (0.02, 0.33)	0.05 (0.01, 0.19)	0.08 (0.02, 0.35)	< 0.001
*Others*					
Median	154.40	218.79	289.44	466.18	
Crude	1	0.07 (0.02, 0.20)	0.05 (0.02, 0.13)	0.02 (0.01, 0.07)	< 0.001
Model 1	1	0.08 (0.02, 0.23)	0.05 (0.02, 0.15)	0.03 (0.01, 0.09)	< 0.001
Model 2	1	0.08 (0.02, 0.23)	0.05 (0.02, 0.15)	0.03 (0.01, 0.09)	< 0.001
Model 3	1	0.06 (0.02, 0.23)	0.06 (0.01, 0.20)	0.02 (0.01, 0.08)	< 0.001
Model 4	1	0.12 (0.03, 0.45)	0.08 (0.02, 0.32)	0.04 (0.01, 0.14)	< 0.001

*Note:* Model 1: Age and sex. Model 2: Model 1 + education, smoking (never/ex‐smoker/current smoker), physical activity (METS/min per week). Model 3: Model 2 + hypercholesterolemia (yes/no), hypertension (yes/no), diabetes mellitus (yes/no), aspirin use (yes/no), family history of cardiovascular disease (yes/no) and body mass index (kg/m^2^). Model 4: Model 3 + intake of energy (kcal/day), legumes, fish, grains, non‐hydrogenated vegetable oils, red meat, sweets, sugar sweetened beverages, dairy, nuts and seeds.

As Table 5 illustrates, in crude model there was an inverse relationship between any increase intake of total fruits and vegetables with the risk of PCAD occurrence in total population (OR: 0.34, 95% CI: 0.25, 0.46, OR: 0.07, 95% CI: 0.05, 0.10 and OR: 0.06, 95% CI: 0.04, 0.07, respectively; *p* for trend < 0.001). This association remained constant to some extent, after adjustment for potential confounders in different models (Model 4: OR: 0.30, 95% CI: 0.21, 0.45, OR: 0.07, 95% CI: 0.05, 0.11 and OR: 0.05, 95% CI: 0.03, 0.07, respectively; *p* for trend < 0.001). In addition, any higher consumption of total fruits and vegetables was related with reducing odds of PCAD diverse ethnicities in which this inverse association was significant.

**TABLE 5:** Odds ratio (95% confidence interval) of premature coronary artery disease risk in different ethnicities according to quartile of total fruits and vegetables consumption.

Ethnicity/Model	OR (95% CI)				*p* for trend
	Q1 < 343.05	343.05 ≤ Q2 < 474.07	474.07 ≤ Q3 < 613.42	Q4 ≥ 613.42	
*Total*					
Median	279.52	402.89	526.93	781.94	
Crude	1	0.34 (0.25, 0.46)	0.07 (0.05, 0.10)	0.06 (0.04, 0.07)	< 0.001
Model 1	1	0.34 (0.25, 0.47)	0.07 (0.05, 0.10)	0.06 (0.04, 0.08)	< 0.001
Model 2	1	0.34 (0.24, 0.46)	0.07 (0.05, 0.10)	0.07 (0.04, 0.08)	< 0.001
Model 3	1	0.31 (0.22, 0.45)	0.07 (0.05, 0.10)	0.06 (0.04, 0.08)	< 0.001
Model 4	1	0.30 (0.21, 0.45)	0.07 (0.05, 0.11)	0.05 (0.03, 0.07)	< 0.001
*Azari*					
Median	263.67	414.36	529.01	769.48	
Crude	1	0.29 (0.09, 0.92)	0.05 (0.01, 0.15)	0.04 (0.01, 0.13)	< 0.001
Model 1	1	0.28 (0.08, 0.90)	0.06 (0.01, 0.18)	0.05 (0.01, 0.15)	< 0.001
Model 2	1	0.27 (0.08, 0.88)	0.06 (0.01, 0.18)	0.05 (0.01, 0.15)	< 0.001
Model 3	1	0.32 (0.08, 1.35)	0.06 (0.02, 0.25)	0.06 (0.01, 0.25)	< 0.001
Model 4	1	0.44 (0.05, 3.69)	0.05 (0.01, 0.45)	0.02 (0.01, 0.28)	< 0.001
*Fars*					
Median	298.49	401.91	528.96	772.39	
Crude	1	0.45 (0.28, 0.70)	0.08 (0.05, 0.13)	0.06 (0.04, 0.09)	< 0.001
Model 1	1	0.48 (0.29, 0.76)	0.09 (0.05, 0.13)	0.07 (0.04, 0.11)	< 0.001
Model 2	1	0.47 (0.29, 0.75)	0.09 (0.05, 0.13)	0.07 (0.04, 0.11)	< 0.001
Model 3	1	0.44 (0.25, 0.78)	0.08 (0.05, 0.14)	0.06 (0.04, 0.11)	< 0.001
Model 4	1	0.40 (0.22, 0.74)	0.09 (0.05, 0.16)	0.06 (0.03, 0.11)	< 0.001
*Gilak*					
Median	231.17	384.07	505.33	664.60	
Crude	1	0.14 (0.06, 0.32)	0.03 (0.01, 0.08)	0.04 (0.01, 0.15)	< 0.001
Model 1	1	0.15 (0.06, 0.36)	0.03 (0.01, 0.08)	0.06 (0.01, 0.21)	< 0.001
Model 2	1	0.17 (0.07, 0.40)	0.03 (0.01, 0.09)	0.05 (0.01, 0.20)	< 0.001
Model 3	1	0.17 (0.06, 0.48)	0.02 (0.01, 0.06)	0.09 (0.02, 0.42)	< 0.001
Model 4	1	0.15 (0.03, 0.77)	0.01 (0.001, 0.05)	0.03 (0.01, 0.34)	< 0.001
*Kurd*					
Median	276.99	407.28	513.76	788.02	
Crude	1	0.24 (0.11, 0.51)	0.04 (0.02, 0.09)	0.03 (0.01, 0.07)	< 0.001
Model 1	1	0.25 (0.11, 0.55)	0.04 (0.01, 0.10)	0.04 (0.01, 0.09)	< 0.001
Model 2	1	0.24 (0.11, 0.53)	0.04 (0.01, 0.10)	0.04 (0.01, 0.08)	< 0.001
Model 3	1	0.23 (0.10, 0.53)	0.05 (0.02, 0.12)	0.04 (0.02, 0.10)	< 0.001
Model 4	1	0.32 (0.11, 0.95)	0.05 (0.01, 0.17)	0.05 (0.01, 0.19)	< 0.001
*Others*					
Median	279.23	400.15	521.07	814.57	
Crude	1	0.36 (0.17, 0.74)	0.11 (0.06, 0.23)	0.06 (0.03, 0.11)	< 0.001
Model 1	1	0.36 (0.16, 0.77)	0.13 (0.06, 0.27)	0.06 (0.03, 0.12)	< 0.001
Model 2	1	0.35 (0.16, 0.77)	0.12 (0.06, 0.26)	0.06 (0.03, 0.12)	< 0.001
Model 3	1	0.29 (0.11, 0.74)	0.10 (0.04, 0.24)	0.05 (0.02, 0.11)	< 0.001
Model 4	1	0.37 (0.13, 1.04)	0.11 (0.04, 0.30)	0.05 (0.02, 0.14)	< 0.001

*Note:* Model 1: Age and sex. Model 2: Model 1 + education, smoking (never/ex‐smoker/current smoker), physical activity (METS/min per week). Model 3: Model 2 + hypercholesterolemia (yes/no), hypertension (yes/no), diabetes mellitus (yes/no), aspirin use (yes/no), family history of cardiovascular disease (yes/no) and body mass index (kg/m^2^). Model 4: Model 3 + intake of energy (kcal/day), legumes, fish, grains, non‐hydrogenated vegetable oils, red meat, sweets, sugar sweetened beverages dairy, nuts and seeds.

The data obtained from Table S1 reveals that there is a markedly inverse relationship between fruit, vegetable, and total fruit and vegetable consumption and the odds of PCAD in both genders (*p* < 0.001). After full adjustment for potential confounders, participants in the 4th quartile of fruit intake had reduced odds of PCAD compared with those in the lowest quartile among males (OR: 0.09; 95% CI: 0.05–0.15) and females (OR: 0.09; 95% CI: 0.06–0.15), respectively. Likewise, the top quartile of vegetable consumption was linked to statistically notable declined odds of PCAD in males ((OR: 0.06; 95% CI: 0.03–0.12); *p* for trend < 0.001) and females (OR: 0.03; 95% CI: 0.01–0.05; *p* for trend < 0.001) in the full adjustment model. Furthermore, the overall intake of fruits and vegetables in both sexes was observed to diminish the risk of PCAD across all models. In the full adjustment model (OR: 0.04, 95% CI: 0.02, 0.09 in males; *p* for trend < 0.001 and OR: 0.05, 95% CI: 0.03, 0.09 in females; *p* for trend < 0.001).

The sensitivity analyses by excluding participants with major cardiovascular risk factors, including diabetes, hypertension, and smoking showed that findings were broadly consistent with those of the primary analysis. After full adjustment for potential confounders, participants in the highest quartile of fruit, vegetables, and total fruit and vegetable intake had reduced odds of PCAD compared with those in the lowest quartile (OR: 0.09, 95% CI: 0.04–0.20; *p* for tend < 0.001; OR: 0.02, 95% CI: 0.01–0.07; *p* for tend < 0.001; OR: 0.05, 95% CI: 0.02–0.11; *p* for tend < 0.001, respectively) (Table S2).

The relationship of fruits, vegetables, and total fruits and vegetables consumption with the severity of PCAD in total population were indicated in Figures 1, 2, 3, respectively. In crude model, compared to the first quartile, the highest quartile of fruits consumption was associated with a 93% reduction in the risk of severe PCAD (OR: 0.07, 95% CI: 0.05, 0.08; *p* < 0.001). After adjustment for potential confounders the result was not changed considerably (OR: 0.07, 95% CI (0.06, 0.10); *p* < 0.001) (Figure 1).

**FIGURE 1:**
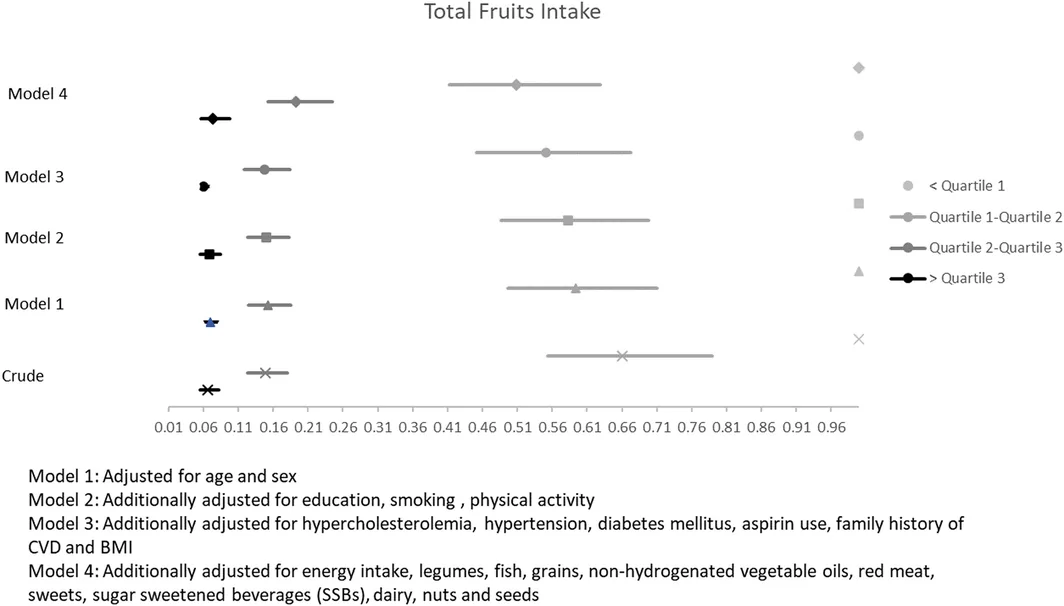
The associations of fruit intake with severity of premature coronary artery disease.

Subjects in the highest quartile of vegetable intake had a decrease in the risk of severe PCAD by 97% (OR: 0.03, 95% CI: 0.02, 0.04; *p* < 0.001) in the crude model and it remained at the same level in the fully adjusted model (OR: 0.05, 95% CI: 0.04, 0.07; *p* < 0.001) (Figure 2).

**FIGURE 2:**
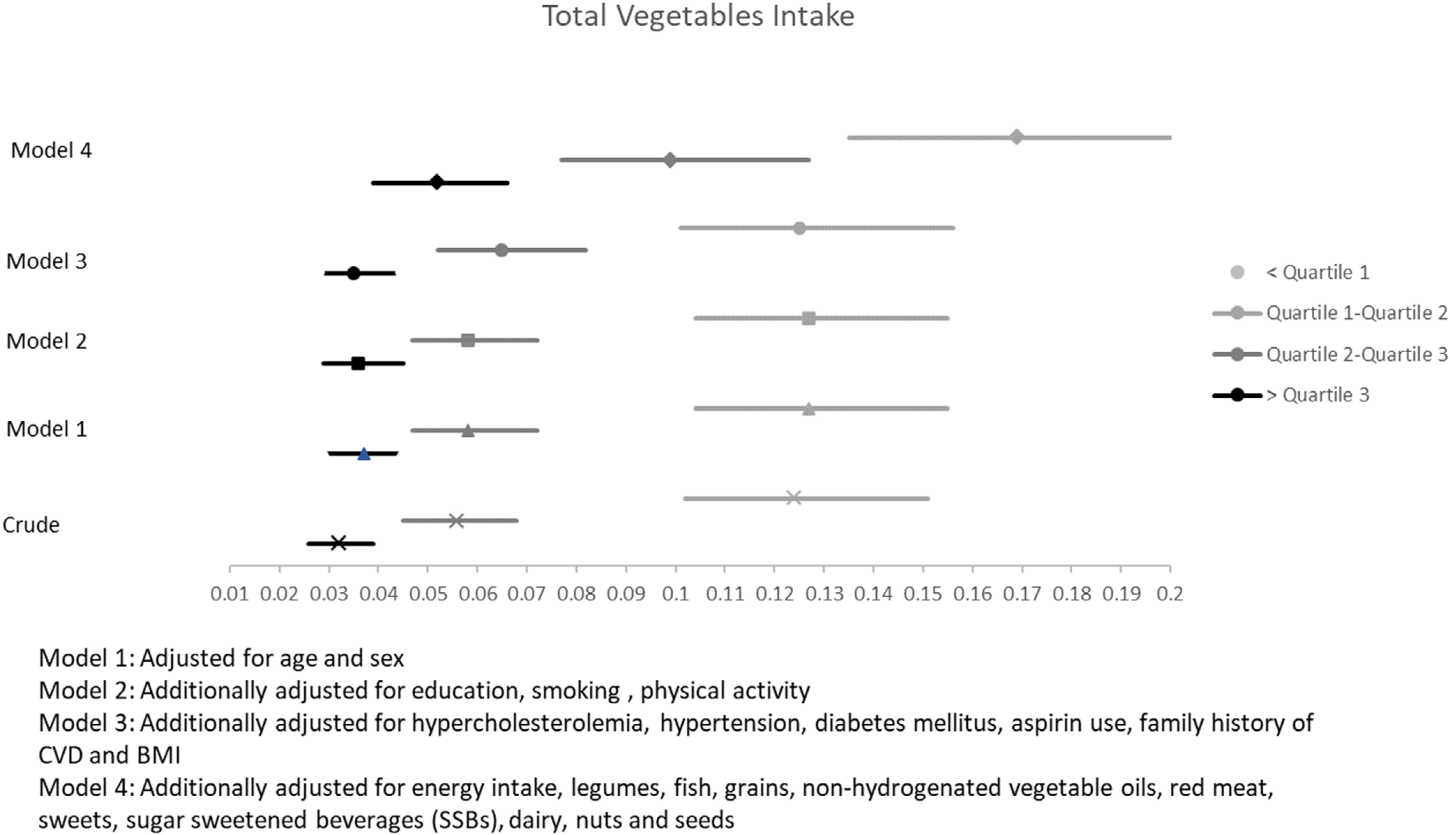
The associations of vegetables intake with severity of premature coronary artery disease.

As Figure 3 shows, the highest quartile of total fruits and vegetables consumption was associated with a 97% reduction in the risk of severe PCAD (OR: 0.03, 95% CI: 0.02, 0.04, *p* < 0.001), while the second quartile only reduced the risk of severe PCAD by 62% (OR: 0.38, 95% CI: 0.32, 0.46, *p* < 0.001). After adjusting for potential confounding variables, the results persist similar (OR: 0.04, 95% CI: 0.02, 0.05; *p* < 0.001).

**FIGURE 3:**
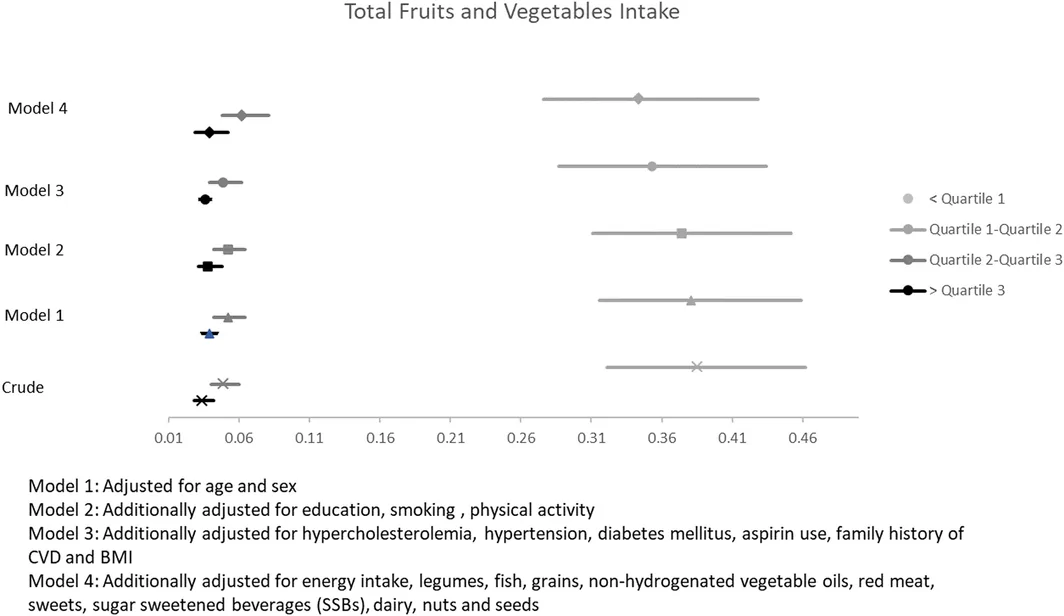
The associations of total fruits and vegetables intake with severity of premature coronary artery disease.

The relationship of fruits, vegetables, and total fruits and vegetables consumption with the severity of PCAD was assessed in male and female separately. Similar results were found for both genders (Figures S1–S6).

## 4 Discussion

This multi‐centric case–control study indicated a significant inverse association between fruits and vegetables consumption and the risk of PCAD. Any increase in intake of fruits, vegetables, and also total fruits and vegetables had an inverse association with PCAD risk. After adjustment for all potential confounding variables, we found that fruits, vegetables, and total fruits and vegetables consumption were associated with 91%, 97%, and 95% risk reduction of PCAD in all ethnicities, respectively. Moreover, there was a significant association between higher consumption of fruits, vegetables, and total of them with reduced risk of severe PCAD.

According to previous studies, there are several risk factors for PCAD, some of which can be modified such as diabetes mellitus, dyslipidemia, hypertension, and smoking (Jayaram and Shah 2015; Poorzand et al. 2019; Sharma et al. 2022). On the other hand, according to a previous study, dietary habits have been evaluated as the most important risk factor for the development of MI in young adults (Panagiotakos et al. 2008). Also, evidence has shown that following inappropriate dietary habits such as high consumption of red meat and processed products, refined grains, and low consumption of fruits and vegetables are more common among healthy young people with a family history of PCAD (Osadnik et al. 2018). On the other hand, the continuation of these unhealthy dietary habits in adulthood may lead to increased cardiovascular risks based on family history (Osadnik et al. 2018). As a result, modification of risk factors, especially revision of dietary habits, can be effective in preventing the development of PCAD.

According to the results of the previous studies, the results of the current studies are in line with most of the available evidence that higher consumption of fruits has been associated with a reduction in the risk of CHD and CVD and its mortality (Miller et al. 2017; Aune et al. 2017; Tayyem et al. 2020; Zurbau et al. 2020). In the meta‐analysis study by Aune et al. conducted in 2017, it has been shown that consumption of 300 g/day of fruits is associated with a lower risk of CHD (Aune et al. 2017). Also, in confirmation of the findings of the current study, a meta‐analysis study conducted in 2020 also showed that higher consumption of fruits is associated with a 9%, 12%, and 18% reduction in the incidence risk of CVD, CHD, and stroke respectively (Zurbau et al. 2020). Although, few studies have not observed such an effect. This might be due to different methodologies and specific cardiovascular diseases, which emphasize the complexity of the diet‐disease relationship and the need for further research across different populations (Yoshizaki et al. 2020). According to previous studies, there are different mechanisms related to reducing the risk of CVD due to higher consumption of fruits. Owing to having high fiber, low glycemic index, suitable nutrients and antioxidants, and also having low calorie density, fruits can manage weight and important risk factors of cardiovascular diseases such as obesity, dyslipidemia, and hypertension (Zurbau et al. 2020; Hartley et al. 2013; Kim and Je 2016; Jenkins et al. 2011; Mytton et al. 2014).

In the case of vegetables, the results of our study confirm the results of other previous studies and the consumption of more vegetables is associated with a reduction in the risk of cardiovascular diseases (Miller et al. 2017; Aune et al. 2017; Zurbau et al. 2020). According to the results of the meta‐analysis study conducted by Zurbau et al., it has been shown that higher consumption of vegetables is related to a decrease in the incidence and mortality of cardiovascular diseases. So that the higher consumption of vegetables reduces the risk of CVD, CHD, and stroke by 6%, 8%, and 12%, respectively (Zurbau et al. 2020). Also, a dose–response meta‐analysis study has shown that consuming 550–600 g/day of vegetables is associated with a 30% reduction in the risk of CHD (Aune et al. 2017). Past evidence indicates that vegetables, due to their high fiber content, antioxidants, vitamins, flavonoids, etc., can reduce the risk of chronic diseases, including CVD, by reducing inflammation, blood pressure, blood cholesterol levels, and regulating weight. Therefore, the consumption of vegetables in the daily diet of people is important (Aune et al. 2017; Bøhn et al. 2010; Anderson et al. 2009; Lampe 1999; Macready et al. 2014).

As mentioned earlier, the results of the present study have revealed that the high co‐consumption of fruits and vegetables is associated with a reduction in the risk of PCAD, which the results of other previous studies on CHD and CVD also confirm the aforementioned results (Miller et al. 2017; Aune et al. 2017; Zurbau et al. 2020). In this regard, a meta‐analysis study conducted in 2020 has shown that the co‐consumption of fruits and vegetables associated with lower the incidence of CVD, CHD, and stroke by 7%–18% and the mortality rate by 11%–27% respectively (Zurbau et al. 2020). Also, in the dose–response meta‐analysis study conducted by Anue et al., it has been indicated that consuming 200 g/day of fruits and vegetables reduces the risk of CVD, CHD, and stroke by 8%–16% (Aune et al. 2017). Although the mechanism of this protective effect of the co‐consumption of fruits and vegetables is not completely clear, past studies have suggested various factors. Fruits and vegetables contain a high density of nutrients, including insoluble and soluble fibers, vitamins, carotenoids, antioxidants, flavonoids, and minerals, which probably reduce the risk of chronic diseases and premature mortality with their synergistic effects (Bøhn et al. 2010; Aune et al. 2017). Dietary fiber is divided into insoluble and soluble fibers determined by solubility. Insoluble fiber, such as plant cellulose and hemicellulose, does not dissolve in water, but soluble fiber, like plant pectin and gums, dissolves in water (Zademohammadi et al. 2024). Insoluble fiber increases stool volume and helps to regulate bowel motions, whereas soluble fibers decrease gastric emptying, speed up small intestine motility, and control nutrient absorption (Theuwissen and Mensink 2008; Nweze et al. 2021). Soluble fiber is hypothesized to minimize the effects of postprandial blood glucose and lipid rises as two main CAD risk factors. Additionally, it is known that both soluble and insoluble fiber enhance stomach distension and affect gut hormones that induce satiety, which results in a decrease in food intake and, in the long time improved glucose metabolism and weight loss (Nweze et al. 2021). Researchers indicate that viscous soluble fibers could impair the metabolism of lipids and/or bile acids, hence lowering serum LDL‐C levels. Other proposed mechanisms include the suppression of hepatic cholesterol synthesis by fermentation products and the delayed absorption of carbohydrates, which leads to enhanced insulin sensitivity (Theuwissen 2008).

It has been shown that fruits and vegetables improve cardiovascular function by reducing cholesterol, blood pressure, inflammation, and platelet aggregation (Anderson et al. 2009; Lampe 1999; Macready et al. 2014; Izadi et al. 2018). An elevated level of oxidative stress and inflammation has been demonstrated to be critical triggers for the initiation and development of CVD. Long‐term inflammatory exposure declines blood antioxidant levels due to the continual formation of a rise in total reactive oxygen species (ROS). ROS may accelerate the oxidation of LDL‐C, cholesterol‐derived species, and protein modification, leading to foam cell production and atherosclerotic plaques in arteries (Zhang et al. 2014). The antioxidants present in fruits and vegetables are indicated to beneficially neutralize the active and damaging species of oxygen (Lampe 1999). Furthermore, the consumption of fruits and vegetables can improve gut microbiota (Luo et al. 2021). The gut microbiota contributes to the production of different short‐chain fatty acids (SCFAs) through dietary intake. The essential function of SCFAs in glucose, lipid, and cholesterol metabolism, as well as immunoregulatory actions, may aid in CVD prevention (Jayachandran et al. 2020).

On the other hand, high consumption of fruits and vegetables instead of unhealthy foods with high calorie density, rich in saturated fat, trans fat, and sodium can be effective in reducing the risk of chronic diseases including cardiovascular diseases (Aune et al. 2017; Paknahad et al. 2020).

The robustness of our findings was further supported by sensitivity analyses excluding participants with diabetes, hypertension, and smoking history. The consistency of the results across these analyses indicates that the observed associations were not substantially influenced by these major cardiovascular risk factors.

Finally, based on our results, we conclude that promoting consumption of fruits and vegetables is suggested as a simple and effective strategy to reduce the risk of PCAD. Informing and educating the society about the importance of combining these two food groups and their health benefits can increase the consumption of fruits and vegetables. Also, promotional programs should be undertaken to encourage people to consume fruits and vegetables at the same time on a daily basis. The very low odds ratios observed for the highest categories of fruit and vegetable intake should be interpreted with caution. While large risk reductions are biologically plausible, particularly when contrasting very low with very high intake levels, residual confounding by other lifestyle or dietary factors and measurement error in dietary assessment may partly contribute to the magnitude of these estimates. Therefore, our findings should be viewed as an evidence for a protective association rather than definitive quantification of the exact effect size.

This study was a unique large study in the Middle East on CAD in adults. An important advantage of our study was the inclusion of diverse Iranian ethnicities from about 14 cities. Therefore, these findings can be generalizable to the Iranian population. We used angiography as the standard for diagnosing CAD in participants. In addition, using a validated semi‐quantitative FFQ for assessment of dietary intakes through in‐person interviews was another advantage of this study. However, our study had some limitations. We utilized an FFQ questionnaire as a memory‐based tool, which may contain some measurement biases. Moreover, selection and recall bias are inevitable in case–control studies. Additionally, there was a significant disparity in the number of men and women included in the sample. Although we applied a validated FFQ to habitual intake during the preceding year and excluded patients with a prior history of CAD or major recent lifestyle changes, the case–control design still makes it possible that subclinical disease or early symptoms may have influenced dietary habits. Thus, temporal ambiguity and reverse causality may remain. Another limitation relates to the use of an FFQ to assess dietary intake, which is subject to recall bias and measurement error; however, our instrument has been validated in the Iranian population and showed acceptable validity and reproducibility for major food groups, including fruits and vegetables. Although we used multivariable‐adjusted effect values to extract associations, residual confounding could not be ruled out.

## 5 Conclusion

In conclusion, the findings of this study support the evidence of the protective impact of fruits and vegetables on the risk of PCAD. Any increase in the consumption of vegetables and also total fruits and vegetables can be useful to reduce the possibility of PCAD. Although there are cultural and nutritional patterns in different ethnicities which emphasize the importance of promoting diverse nutritional patterns, our findings support the role of increased fruit and vegetable consumption in reducing the risk of PCAD. Clinicians should emphasize dietary interventions promoting higher intake of fruits and vegetables, particularly for at‐risk individuals. Early education and public health initiatives to improve access to fresh produce are also recommended. These strategies can be integrated into cardiovascular risk management to help prevent PCAD. Further well‐designed randomized clinical trials are suggested to strengthen these findings and to better understand the mechanisms involved in this process.

## Author Contributions

**Noushin Mohammadifard:** investigation, writing – original draft. **Sara Aghakhani:** data curation, investigation. **Ehsan Zarepur:** methodology, investigation. **Alireza Khosravi Farsani:** formal analysis, data curation. **Nahid Azdaki:** data curation, methodology. **Nahid Salehi:** investigation, data curation. **Masoud Lotfizadeh:** data curation. **Samad Ghaffari:** methodology, investigation. **Arsalan Salari:** formal analysis, data curation. **Mostafa Cheraghi:** data curation, investigation. **Ahmadreza Assareh:** data curation. **Razieh Hassannejad:** writing – review and editing, validation. **Azam Soleimani:** writing – review and editing, supervision. **Nizal Sarrafzadegan:** conceptualization.

## Funding

This study was funded by the Research and Technology Department, Iran Ministry of Health and Medical Education, and Iranian Network of Cardiovascular Research (grant number: 96110).

## Conflicts of Interest

The authors declare no conflicts of interest.

## Supporting information


**Table S1:** Odds ratio (95% confidence interval) of premature coronary artery disease risk in gender according to quartile of fruits, vegetables and total fruits and vegetables consumption.
**Table S2:** Odds ratio (95% confidence interval) of premature coronary artery disease risk according to quartile of fruits, vegetables and total fruits and vegetables consumption by excluding participants with major cardiovascular risk factors, including diabetes, hypertension, and smoking.
**Figure S1:** The associations of total fruits intake in male with severity of premature coronary artery disease.
**Figure S2:** The associations of total vegetables intake in male with severity of premature coronary artery disease.
**Figure S3:** The associations of total fruits and vegetables intake in male with severity of premature coronary artery disease.
**Figure S4:** The associations of total fruits intake in female with severity of premature coronary artery disease.
**Figure S5:** The associations of total vegetables intake in female with severity of premature coronary artery disease.
**Figure S6:** The associations of total fruits and vegetables intake in female with severity of premature coronary artery disease.

## Data Availability

The data that support the findings of this study are available on request from the corresponding author.
