# Supplementary material for: Association Between Fruits and Vegetables Consumption and Premature Coronary Artery Disease (PCAD): A Multi‐Center Case Control Study
Source: Food Sci Nutr. 2026 Sep 29;14(10):e72371. doi: 10.1002/fsn3.72371 (PMC13620836; doi:10.1002/fsn3.72371)
Supplement: Supplementary file 1 [file FSN3-14-e72371-s001.docx]

**Supplementary Table 1- Odds ratio (95% confidence interval) of premature coronary artery disease risk in gender according to quartile of fruits, vegetables and total fruits and vegetables consumption**

|  | **Fruits** | | | | **Vegetables** | | | | **Total** | | | |
| --- | --- | --- | --- | --- | --- | --- | --- | --- | --- | --- | --- | --- |
| **Male:** | **Q1 <150.08** | **150.08 ≤Q2< 205.28** | **205.28≤Q3< 275.70** | **Q4 ≥ 275.70** | **Q1 <183.72** | **183.72≤Q2< 243.59** | **243.59≤Q3< 319.21** | **Q4 ≥ 319.21** | **Q1 <337.07** | **337.07≤Q2< 450.56** | **450.56≤Q3< 593.88** | **Q4 ≥ 593.88** |
| **Median** | 94.88 | 178.37 | 242.88 | 339.73 | 156.21 | 222.44 | 286.32 | 440.84 | 284.82 | 407.57 | 531.23 | 758.74 |
| **Crude** | 1 | 1.09  (0.69, 1.74) | 0.20  (0.14, 0.31) | 0.09  (0.06, 0.13) | 1 | 0.07  (0.04, 0.14) | 0.05 (0.02, 0.09) | 0.03  (0.02, 0.06) | 1 | 0.29  (0.17, 0.50) | 0.06  (0.03, 0.10) | 0.04  (0.02, 0.07) |
| **Model 1** | 1 | 1.11  (0.69, 1.78) | 0.23  (0.15, 0.34) | 0.11  (0.07, 0.16) | 1 | 0.08  (0.04, 0.14) | 0.05  (0.02, 0.09) | 0.04  (0.02, 0.07) | 1 | 0.32  (0.19, 0.55) | 0.07  (0.04, 0.12) | 0.05  (0.03, 0.09) |
| **Model 2** | 1 | 1.09  (0.68, 1.75) | 0.23  (0.15, 0.34) | 0.10  (0.07, 0.16) | 1 | 0.08  (0.04, 0.14) | 0.05  (0.02, 0.09) | 0.04  (0.02, 0.07) | 1 | 0.32  (0.18, 0.55) | 0.07  (0.04, 0.12) | 0.05  (0.03, 0.09) |
| **Model 3** | 1 | 0.95  (0.57, 1.59) | 0.24  (0.15, 0.37) | 0.10  (0.06, 0.16) | 1 | 0.08  (0.04, 0.15) | 0.06  (0.03, 0.11) | 0.05  (0.02, 0.09) | 1 | 0.33  (0.18, 0.59) | 0.07  (0.04, 0.12) | 0.06  (0.03, 0.10) |
| **Model 4** | 1 | 0.91  (0.51, 1.60) | 0.23  (0.14, 0.40) | 0.09  (0.05, 0.15) | 1 | 0.10  (0.05, 0.20) | 0.08  (0.04, 0.16) | 0.06  (0.03, 0.12) | 1 | 0.35  (0.18, 0.66) | 0.08 (0.04, 0.15) | 0.04  (0.02, 0.09) |
| **Female:** | **Q1 <139.49** | **139.49≤Q2< 216.60** | **216.60≤Q3< 283.08** | **Q4 ≥ 283.08** | **Q1 <205.26** | **205.26≤Q2< 271.40** | **271.40≤Q3< 365.07** | **Q4 ≥ 365.07** | **Q1 <351.84** | **351.84≤Q2< 498.99** | **498.99≤Q3< 635.56** | **Q4 ≥ 635.56** |
| **Median** | 86.29 | 180.96 | 238.96 | 346.45 | 158.73 | 224.36 | 292.56 | 438.03 | 270.52 | 409.39 | 542.92 | 746.02 |
| **Crude** | 1 | 0.78  (0.56, 1.08) | 0.22  (0.16, 0.30) | 0.09  (0.07, 0.13) | 1 | 0.05  (0.03, 0.10) | 0.03  (0.01, 0.05) | 0.02  (0.01, 0.03) | 1 | 0.32  (0.22, 0.47) | 0.06 (0.04, 0.10) | 0.05  (0.03, 0.08) |
| **Model 1** | 1 | 0.84  (0.59, 1.17) | 0.24  (0.18, 0.33) | 0.11  (0.08, 0.16) | 1 | 0.06  (0.03, 0.10) | 0.03  (0.01, 0.05) | 0.02  (0.01, 0.04) | 1 | 0.35  (0.23, 0.51) | 0.07 (0.05, 0.11) | 0.06  (0.04, 0.10) |
| **Model 2** | 1 | 0.83  (0.59, 1.17) | 0.24  (0.17, 0.33) | 0.11  (0.07, 0.15) | 1 | 0.06  (0.03, 0.10) | 0.03  (0.01, 0.05) | 0.02  (0.01, 0.04) | 1 | 0.34  (0.23, 0.51) | 0.07 (0.05, 0.10) | 0.06  (0.04, 0.09) |
| **Model 3** | 1 | 0.84 (0.56,1.25) | 0.24  (0.16, 0.34) | 0.10  (0.07, 0.15) | 1 | 0.05  (0.03,0.09) | 0.03  (0.02, 0.06) | 0.02  (0.01, 0.04) | 1 | 0.30  (0.19,0.48) | 0.06 (0.04, 0.10) | 0.06  (0.04, 0.10) |
| **Model 4** | 1 | 0.79  (0.50, 1.24) | 0.22  (0.15, 0.34) | 0.09  (0.06, 0.15) | 1 | 0.06  (0.03, 0.12) | 0.04  (0.02, 0.8 | 0.03  (0.01, 0.05) | 1 | 0.28  (0.17, 0.46) | 0.06  (0.04, 0.10) | 0.05  (0.03, 0.09) |

All P for tends were <0.001

Model 1: Age

Model 2: Model 1 + education, smoking (never/ ex-smoker/ current smoker), physical activity (METS/ minute per week)

Model 3: Model 2 + hypercholesterolemia (yes/ no), hypertension (yes/ no), diabetes mellitus (yes/ no), aspirin use (yes/ no), family history of cardiovascular disease (yes/ no) and body mass index (Kg/ m^2^)

Model 4: Model 3 + intake of energy (Kcal/ day), legumes, fish, grains, non-hydrogenated vegetable oils, red meat, sweets, sugar sweetened beverages, dairy, nuts and seeds.

**Supplementary Table 2- Odds ratio (95% confidence interval) of premature coronary artery disease risk according to quartile of fruits, vegetables and total fruits and vegetables consumption by excluding participants with major cardiovascular risk factors, including diabetes, hypertension, and smoking**

|  | **Fruits** | | | | **Vegetables** | | | | **Total** | | | |
| --- | --- | --- | --- | --- | --- | --- | --- | --- | --- | --- | --- | --- |
|  | **Q1 <163.99** | **163.99 ≤Q2< 230.48** | **230.48 ≤Q3< 297.98** | **Q4 ≥ 297.98** | **Q1 <209.69** | **209.69≤Q2< 277.01** | **277.01≤Q3< 361.61** | **Q4 ≥ 361.61** | **Q1 <378.96** | **378.96 ≤Q2< 519.04** | **519.04≤Q3< 639.64** | **Q4 ≥ 639.64** |
| **Median** | 87.43 | 176.79 | 240.53 | 338.22 | 157.01 | 220.71 | 285.81 | 453.40 | 279.52 | 402.89 | 526.93 | 781.94 |
| **Crude** | 1 | 0.89  (0.53, 1.50) | 0.27  0.17, 0.43) | 0.13  (0.08, 0.21) | 1 | 0.07  (0.03, 0.13) | 0.05  (0.02, 0.09) | 0.03  (0.02, 0.07) | 1 | 0.43  (0.25, 0.76) | 0.11  (0.07, 0.19) | 0.07  (0.04,0.13) |
| **Model 1** | 1 | 0.72  (0.41, 1.26) | 0.25  (0.15, 0.42) | 0.1  (0.07, 0.19) | 1 | 0.06  (0.03, 0.13) | 0.05  (0.02, 0.10) | 0.04  (0.02, 0.08) | 1 | 0.48  (0.26, 0.87) | 0.10  (0.06, 0.18) | 0.08  (0.04,0.14) |
| **Model 2** | 1 | 0.71  (0.40, 1.24) | 0.25  (0.15, 0.41) | 0.11  (0.06, 0.19) | 1 | 0.06  (0.03, 0.13) | 0.05  (0.02, 0.10) | 0.04  (0.02 0.08) | 1 | 0.47  (0.25, 0.85) | 0.10  (0.06, 0.18) | 0.08  (0.04, 0.14) |
| **Model 3** | 1 | 0.81  0.42, 1.54) | 0.23  (0.13, 0.42) | 0.11  (0.06, 0.20) | 1 | 0.05  (0.02, 0.11) | 0.04  (0.02, 0.10) | 0.03  (0.01, 0.07) | 1 | 0.51  (0.25, 1.01) | 0.10  (0.05, 0.19) | 0.07  (0.03, 0.13) |
| **Model 4** | 1 | 0.72  (0.34, 1.51) | 0.21  (0.11, 0.40) | 0.09  (0.04, 0.20) | 1 | 0.05  (0.02, 0.12) | 0.04  (0.02, 0.11) | 0.02  (0.01, 0.07) | 1 | 0.43  (0.20, 0.93) | 0.10  (0.05, 0.20) | 0.05  (0.02, 0.11) |

All P for tends were <0.001

Model 1: Age and sex

Model 2: Model 1 + education, physical activity (METS/ minute per week)

Model 3: Model 2 + hypercholesterolemia (yes/ no), aspirin use (yes/ no), family history of cardiovascular disease (yes/ no) and body mass index (Kg/ m^2^)

Model 4: Model 3 + intake of energy (Kcal/ day), legumes, fish, grains, non-hydrogenated vegetable oils, red meat, sweets, sugar sweetened beverages, dairy, nuts and seeds.

Male Total Fruits Intake

| Model 2 | < Quartile 1 |
| --- | --- |
| 1 | 16 |
| 0 | 16 |
| 0 | 16 |

| Model 2 | Quartile 1-Quartile 2 |
| --- | --- |
| 0.52 | 15 |
| 0.4 | 15 |
| 0.677 | 15 |

| Model 2 | Quartile 2-Quartile 3 |
| --- | --- |
| 0.115 | 14 |
| 0.084 | 14 |
| 0.156 | 14 |

| Model 2 |  |
| --- | --- |
| 0.187 | 18 |
| 0.134 | 18 |
| 0.261 | 18 |

| Model 2 |  |
| --- | --- |
| 0.51 | 19 |
| 0.385 | 19 |
| 0.676 | 19 |

| Model 2 |  |
| --- | --- |
| 1 | 20 |
| 0 | 20 |
| 0 | 20 |

| Model 2 |  |
| --- | --- |
| 0.572 | 11 |
| 0.451 | 11 |
| 0.726 | 11 |

| Model 2 |  |
| --- | --- |
| 1 | 12 |
| 0 | 12 |
| 0 | 12 |

| Model 2 |  |
| --- | --- |
| 0.588 | 7 |
| 0.464 | 7 |
| 0.745 | 7 |

| Model 2 |  |
| --- | --- |
| 1 | 8 |
| 0 | 8 |
| 0 | 8 |

| Model 2 | > Quartile 3 |
| --- | --- |
| 0.045 | 13 |
| 0.032 | 13 |
| 0.062 | 13 |

| Model 2 | > Quartile 3 |
| --- | --- |
| 0.068 | 17 |
| 0.046 | 17 |
| 0.098 | 17 |

| Model 2 | > Quartile 3 |
| --- | --- |
| 0.045 | 13 |
| 0.032 | 13 |
| 0.062 | 13 |

| Model 2 | Quartile 2-Quartile 3 |
| --- | --- |
| 0.119 | 10 |
| 0.09 | 10 |
| 0.157 | 10 |

| Model 2 | > Quartile 3 |
| --- | --- |
| 0.052 | 9 |
| 0.039 | 9 |
| 0.069 | 9 |

| Model 2 | Quartile 2-Quartile 3 |
| --- | --- |
| 0.121 | 6 |
| 0.092 | 6 |
| 0.16 | 6 |

| Model 2 | > Quartile 3 |
| --- | --- |
| 0.053 | 5 |
| 0.039 | 5 |
| 0.07 | 5 |

| Model 2 | < Quartile 1 |
| --- | --- |
| 1 | 4 |
| 0 | 4 |
| 0 | 4 |

| Model 2 | Quartile 2-Quartile 3 |
| --- | --- |
| 0.112 | 2 |
| 0.085 | 2 |
| 0.148 | 2 |

| Model 2 | > Quartile 3 |
| --- | --- |
| 0.046 | 1 |
| 0.034 | 1 |
| 0.061 | 1 |

**Supplementary Figure 1- The associations of total fruits intake in male with severity of premature coronary artery disease**

Male Total Vegetables Intake

| Model 2 | < Quartile 1 |
| --- | --- |
| 1 | 16 |
| 0 | 16 |
| 0 | 16 |

| Model 2 | Quartile 1-Quartile 2 |
| --- | --- |
| 0.144 | 15 |
| 0.109 | 15 |
| 0.191 | 15 |

| Model 2 | Quartile 2-Quartile 3 |
| --- | --- |
| 0.065 | 14 |
| 0.048 | 14 |
| 0.088 | 14 |

| Model 2 |  |
| --- | --- |
| 0.122 | 18 |
| 0.088 | 18 |
| 0.169 | 18 |

| Model 2 |  |
| --- | --- |
| 0.2 | 19 |
| 0.149 | 19 |
| 0.269 | 19 |

| Model 2 |  |
| --- | --- |
| 1 | 20 |
| 0 | 20 |
| 0 | 20 |

| Model 2 |  |
| --- | --- |
| 0.147 | 11 |
| 0.114 | 11 |
| 0.19 | 11 |

| Model 2 |  |
| --- | --- |
| 1 | 12 |
| 0 | 12 |
| 0 | 12 |

| Model 2 |  |
| --- | --- |
| 0.146 | 7 |
| 0.113 | 7 |
| 0.188 | 7 |

| Model 2 |  |
| --- | --- |
| 1 | 8 |
| 0 | 8 |
| 0 | 8 |

| Model 2 | > Quartile 3 |
| --- | --- |
| 0.038 | 13 |
| 0.027 | 13 |
| 0.052 | 13 |

| Model 2 | > Quartile 3 |
| --- | --- |
| 0.07 | 17 |
| 0.048 | 17 |
| 0.103 | 17 |

| Model 2 | > Quartile 3 |
| --- | --- |
| 0.038 | 13 |
| 0.027 | 13 |
| 0.052 | 13 |

| Model 2 | Quartile 2-Quartile 3 |
| --- | --- |
| 0.064 | 10 |
| 0.049 | 10 |
| 0.084 | 10 |

| Model 2 | > Quartile 3 |
| --- | --- |
| 0.038 | 9 |
| 0.028 | 9 |
| 0.05 | 9 |

| Model 2 | Quartile 2-Quartile 3 |
| --- | --- |
| 0.063 | 6 |
| 0.048 | 6 |
| 0.083 | 6 |

| Model 2 | > Quartile 3 |
| --- | --- |
| 0.037 | 5 |
| 0.028 | 5 |
| 0.05 | 5 |

| Model 2 | < Quartile 1 |
| --- | --- |
| 1 | 4 |
| 0 | 4 |
| 0 | 4 |

| Model 2 | Quartile 2-Quartile 3 |
| --- | --- |
| 0.062 | 2 |
| 0.048 | 2 |
| 0.081 | 2 |

| Model 2 | > Quartile 3 |
| --- | --- |
| 0.035 | 1 |
| 0.026 | 1 |
| 0.046 | 1 |

**Supplementary Figure 2- The associations of total vegetables intake in male with severity of premature coronary artery disease**

Male Total Fruits and Vegetables Intake

| Model 2 | < Quartile 1 |
| --- | --- |
| 1 | 16 |
| 0 | 16 |
| 0 | 16 |

| Model 2 | Quartile 1-Quartile 2 |
| --- | --- |
| 0.386 | 15 |
| 0.296 | 15 |
| 0.504 | 15 |

| Model 2 | Quartile 2-Quartile 3 |
| --- | --- |
| 0.042 | 14 |
| 0.03 | 14 |
| 0.058 | 14 |

| Model 2 |  |
| --- | --- |
| 0.066 | 18 |
| 0.046 | 18 |
| 0.095 | 18 |

| Model 2 |  |
| --- | --- |
| 0.391 | 19 |
| 0.294 | 19 |
| 0.52 | 19 |

| Model 2 |  |
| --- | --- |
| 1 | 20 |
| 0 | 20 |
| 0 | 20 |

| Model 2 |  |
| --- | --- |
| 0.418 | 11 |
| 0.329 | 11 |
| 0.532 | 11 |

| Model 2 |  |
| --- | --- |
| 1 | 12 |
| 0 | 12 |
| 0 | 12 |

| Model 2 |  |
| --- | --- |
| 0.428 | 7 |
| 0.337 | 7 |
| 0.543 | 7 |

| Model 2 |  |
| --- | --- |
| 1 | 8 |
| 0 | 8 |
| 0 | 8 |

| Model 2 | > Quartile 3 |
| --- | --- |
| 0.028 | 13 |
| 0.02 | 13 |
| 0.04 | 13 |

| Model 2 | > Quartile 3 |
| --- | --- |
| 0.037 | 17 |
| 0.025 | 17 |
| 0.056 | 17 |

| Model 2 | > Quartile 3 |
| --- | --- |
| 0.028 | 13 |
| 0.02 | 13 |
| 0.04 | 13 |

| Model 2 | Quartile 2-Quartile 3 |
| --- | --- |
| 0.045 | 10 |
| 0.034 | 10 |
| 0.061 | 10 |

| Model 2 | > Quartile 3 |
| --- | --- |
| 0.03 | 9 |
| 0.022 | 9 |
| 0.041 | 9 |

| Model 2 | Quartile 2-Quartile 3 |
| --- | --- |
| 0.045 | 6 |
| 0.034 | 6 |
| 0.061 | 6 |

| Model 2 | > Quartile 3 |
| --- | --- |
| 0.031 | 5 |
| 0.023 | 5 |
| 0.041 | 5 |

| Model 2 | < Quartile 1 |
| --- | --- |
| 1 | 4 |
| 0 | 4 |
| 0 | 4 |

| Model 2 | Quartile 2-Quartile 3 |
| --- | --- |
| 0.042 | 2 |
| 0.031 | 2 |
| 0.056 | 2 |

| Model 2 | > Quartile 3 |
| --- | --- |
| 0.027 | 1 |
| 0.02 | 1 |
| 0.036 | 1 |

**Supplementary Figure 3- The associations of total fruits and vegetables intake in male with severity of premature coronary artery disease**

Female Total Fruits Intake

| Model 2 | < Quartile 1 |
| --- | --- |
| 1 | 16 |
| 0 | 16 |
| 0 | 16 |

| Model 2 | Quartile 1-Quartile 2 |
| --- | --- |
| 0.633 | 15 |
| 0.466 | 15 |
| 0.86 | 15 |

| Model 2 | Quartile 2-Quartile 3 |
| --- | --- |
| 0.195 | 14 |
| 0.142 | 14 |
| 0.268 | 14 |

| Model 2 |  |
| --- | --- |
| 0.213 | 18 |
| 0.151 | 18 |
| 0.302 | 18 |

| Model 2 |  |
| --- | --- |
| 0.569 | 19 |
| 0.409 | 19 |
| 0.792 | 19 |

| Model 2 |  |
| --- | --- |
| 1 | 20 |
| 0 | 20 |
| 0 | 20 |

| Model 2 |  |
| --- | --- |
| 0.645 | 11 |
| 0.49 | 11 |
| 0.85 | 11 |

| Model 2 |  |
| --- | --- |
| 1 | 12 |
| 0 | 12 |
| 0 | 12 |

| Model 2 |  |
| --- | --- |
| 0.649 | 7 |
| 0.493 | 7 |
| 0.854 | 7 |

| Model 2 |  |
| --- | --- |
| 1 | 8 |
| 0 | 8 |
| 0 | 8 |

| Model 2 | > Quartile 3 |
| --- | --- |
| 0.075 | 13 |
| 0.052 | 13 |
| 0.108 | 13 |

| Model 2 | > Quartile 3 |
| --- | --- |
| 0.074 | 17 |
| 0.049 | 17 |
| 0.112 | 17 |

| Model 2 | > Quartile 3 |
| --- | --- |
| 0.075 | 13 |
| 0.052 | 13 |
| 0.108 | 13 |

| Model 2 | Quartile 2-Quartile 3 |
| --- | --- |
| 0.191 | 10 |
| 0.144 | 10 |
| 0.254 | 10 |

| Model 2 | > Quartile 3 |
| --- | --- |
| 0.08 | 9 |
| 0.058 | 9 |
| 0.111 | 9 |

| Model 2 | Quartile 2-Quartile 3 |
| --- | --- |
| 0.193 | 6 |
| 0.145 | 6 |
| 0.256 | 6 |

| Model 2 | > Quartile 3 |
| --- | --- |
| 0.084 | 5 |
| 0.06 | 5 |
| 0.115 | 5 |

| Model 2 | < Quartile 1 |
| --- | --- |
| 1 | 4 |
| 0 | 4 |
| 0 | 4 |

| Model 2 | Quartile 2-Quartile 3 |
| --- | --- |
| 0.173 | 2 |
| 0.13 | 2 |
| 0.229 | 2 |

| Model 2 | > Quartile 3 |
| --- | --- |
| 0.071 | 1 |
| 0.052 | 1 |
| 0.097 | 1 |

**Supplementary Figure 4- The associations of total fruits intake in female with severity of premature coronary artery disease**

Female Total Vegetables Intake

| Model 2 | < Quartile 1 |
| --- | --- |
| 1 | 16 |
| 0 | 16 |
| 0 | 16 |

| Model 2 | Quartile 1-Quartile 2 |
| --- | --- |
| 0.112 | 15 |
| 0.082 | 15 |
| 0.153 | 15 |

| Model 2 | Quartile 2-Quartile 3 |
| --- | --- |
| 0.052 | 14 |
| 0.037 | 14 |
| 0.072 | 14 |

| Model 2 |  |
| --- | --- |
| 0.064 | 18 |
| 0.044 | 18 |
| 0.092 | 18 |

| Model 2 |  |
| --- | --- |
| 0.111 | 19 |
| 0.078 | 19 |
| 0.157 | 19 |

| Model 2 |  |
| --- | --- |
| 1 | 20 |
| 0 | 20 |
| 0 | 20 |

| Model 2 |  |
| --- | --- |
| 0.147 | 11 |
| 0.114 | 11 |
| 0.19 | 11 |

| Model 2 |  |
| --- | --- |
| 1 | 12 |
| 0 | 12 |
| 0 | 12 |

| Model 2 |  |
| --- | --- |
| 0.113 | 7 |
| 0.083 | 7 |
| 0.155 | 7 |

| Model 2 |  |
| --- | --- |
| 1 | 8 |
| 0 | 8 |
| 0 | 8 |

| Model 2 | > Quartile 3 |
| --- | --- |
| 0.034 | 13 |
| 0.025 | 13 |
| 0.048 | 13 |

| Model 2 | > Quartile 3 |
| --- | --- |
| 0.033 | 17 |
| 0.022 | 17 |
| 0.048 | 17 |

| Model 2 | > Quartile 3 |
| --- | --- |
| 0.034 | 13 |
| 0.025 | 13 |
| 0.048 | 13 |

| Model 2 | Quartile 2-Quartile 3 |
| --- | --- |
| 0.064 | 10 |
| 0.049 | 10 |
| 0.084 | 10 |

| Model 2 | > Quartile 3 |
| --- | --- |
| 0.038 | 9 |
| 0.028 | 9 |
| 0.05 | 9 |

| Model 2 | Quartile 2-Quartile 3 |
| --- | --- |
| 0.052 | 6 |
| 0.037 | 6 |
| 0.073 | 6 |

| Model 2 | > Quartile 3 |
| --- | --- |
| 0.036 | 5 |
| 0.025 | 5 |
| 0.05 | 5 |

| Model 2 | < Quartile 1 |
| --- | --- |
| 1 | 4 |
| 0 | 4 |
| 0 | 4 |

| Model 2 | Quartile 2-Quartile 3 |
| --- | --- |
| 0.045 | 2 |
| 0.033 | 2 |
| 0.063 | 2 |

| Model 2 | > Quartile 3 |
| --- | --- |
| 0.029 | 1 |
| 0.021 | 1 |
| 0.041 | 1 |

**Supplementary Figure 5- The associations of total vegetables intake in female with severity of premature coronary artery disease**

Female Total Fruits and Vegetables Intake

| Model 2 | < Quartile 1 |
| --- | --- |
| 1 | 16 |
| 0 | 16 |
| 0 | 16 |

| Model 2 | Quartile 1-Quartile 2 |
| --- | --- |
| 0.349 | 15 |
| 0.252 | 15 |
| 0.483 | 15 |

| Model 2 | Quartile 2-Quartile 3 |
| --- | --- |
| 0.054 | 14 |
| 0.038 | 14 |
| 0.077 | 14 |

| Model 2 |  |
| --- | --- |
| 0.053 | 18 |
| 0.036 | 18 |
| 0.079 | 18 |

| Model 2 |  |
| --- | --- |
| 0.329 | 19 |
| 0.233 | 19 |
| 0.465 | 19 |

| Model 2 |  |
| --- | --- |
| 1 | 20 |
| 0 | 20 |
| 0 | 20 |

| Model 2 |  |
| --- | --- |
| 0.357 | 11 |
| 0.267 | 11 |
| 0.479 | 11 |

| Model 2 |  |
| --- | --- |
| 1 | 12 |
| 0 | 12 |
| 0 | 12 |

| Model 2 |  |
| --- | --- |
| 0.36 | 7 |
| 0.269 | 7 |
| 0.483 | 7 |

| Model 2 |  |
| --- | --- |
| 1 | 8 |
| 0 | 8 |
| 0 | 8 |

| Model 2 | > Quartile 3 |
| --- | --- |
| 0.044 | 13 |
| 0.03 | 13 |
| 0.064 | 13 |

| Model 2 | > Quartile 3 |
| --- | --- |
| 0.039 | 17 |
| 0.025 | 17 |
| 0.061 | 17 |

| Model 2 | > Quartile 3 |
| --- | --- |
| 0.044 | 13 |
| 0.03 | 13 |
| 0.064 | 13 |

| Model 2 | Quartile 2-Quartile 3 |
| --- | --- |
| 0.057 | 10 |
| 0.041 | 10 |
| 0.078 | 10 |

| Model 2 | > Quartile 3 |
| --- | --- |
| 0.047 | 9 |
| 0.034 | 9 |
| 0.065 | 9 |

| Model 2 | Quartile 2-Quartile 3 |
| --- | --- |
| 0.058 | 6 |
| 0.042 | 6 |
| 0.08 | 6 |

| Model 2 | > Quartile 3 |
| --- | --- |
| 0.048 | 5 |
| 0.034 | 5 |
| 0.066 | 5 |

| Model 2 | < Quartile 1 |
| --- | --- |
| 1 | 4 |
| 0 | 4 |
| 0 | 4 |

| Model 2 | Quartile 2-Quartile 3 |
| --- | --- |
| 0.052 | 2 |
| 0.038 | 2 |
| 0.071 | 2 |

| Model 2 | > Quartile 3 |
| --- | --- |
| 0.039 | 1 |
| 0.028 | 1 |
| 0.054 | 1 |

**Supplementary Figure 6- The associations of total fruits and vegetables intake in female with severity of premature coronary artery disease**
